# 

**Published:** 1891-10

**Authors:** 


CONTENTS.

Vol. 38. No. 10.

| | PAGE. |
|---|---|
| Executions by Electricity ..... | 217 |
| Cheese as an Article of Diet.... | 218 |
| Youth and Old Age .... ...... | 219 |
| Exercise for Girls. . .......... | 220 |
| Inflamed Eyelids.............. | 221 |
| Hay Fever ..................... | 222 |
| The Hair ...................... | 223 |
| How to Grow Plump ... ...... | 224 |
| Short and Tall Men . ....... .... | 224 |
| How to Speak .......... ..... | 225 |
| Table Manners.... ..... . ... | 226 |
| Austrian Women ............. | 227 |
| Eucalyptus Disinfectant ..... | 228 |
| Marriage among New York Plutocrats .............. ....... | 229 |
| Against Alcohol ............. | 230 |
| Cure for Round Shoulders ..... | 232 |
| Ammonia as a Power .... .... | 232 |
| Electric Belts and Pads ........ | 233 |
| Alcohol in Patent Medicines... | 234 |
| A Dying Wife's Dream .. ..... | 235 |
| Miscellaneous ...... .... .. . | 236 |
| How to Cook a Husband .. .... | 238 |
| Food Exhibition ... ......... | 239 |
| Literary . ........ .... .... | 240 |
| Weary ..... .. . ........ .... | 240 |

INDEX TO ADVERTISEMENTS OF HALF A PAGE AND OVER.

| | PAGE. |
|---|---|
| Horsford's Acid Phosphate... | I |
| Lydia E. Pinkham Med. Co.. | II |
| Erie Medical Co.............. | III |
| Bovinine ...... ............... | IV |
| Ruckel & Hendel....... ..... | V |
| Dr. J. C. Ayer & Co ......... | V |
| Kahn Tailoring Co. .... ...... | VIII |
| Herba Vita.................. | VIII |
| Hall's Journal Premiums.. ... | IX |
| Rochester Lamp Co.... ... .. | X |
| Royal Baking Powder........ | XI |
| I. S. Johnson & Co........... | XI |

